# Shape-Controlled Synthesis of Cu_3_TeO_6_ Nanoparticles with Photocatalytic Features

**DOI:** 10.1021/acs.cgd.3c00929

**Published:** 2023-11-07

**Authors:** Javier Fernández-Catalá, Laura Jussila, Matyas Daboczi, Filipp Temerov, Salvador Eslava, Rossella Greco, Wei Cao

**Affiliations:** † Nano and Molecular Systems Research Unit, University of Oulu, Oulu FIN-90014, Finland; ‡ Materials Institute and Inorganic Chemistry Department, University of Alicante, Ap. 99, Alicante E-03080, Spain; § Department of Chemical Engineering and Centre for Processable Electronics, Imperial College London, London SW7 2AZ, U.K.

## Abstract

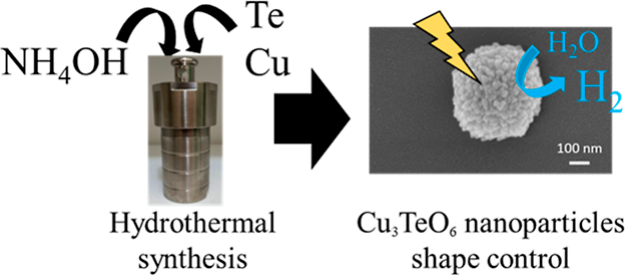

Cu_3_TeO_6_ (CTO) has been synthesized by hydrothermal synthesis applying different pH values without any template or a calcination step to control the crystalline phase and the morphology of nanoparticles. The physicochemical properties characterized by X-ray diffraction, field emission scanning electron microscopy, transmission electron microscopy, N_2_ adsorption, X-ray photoelectron spectroscopy, and diffuse reflectance ultraviolet–visible (DRUV–vis) spectroscopy techniques revealed that the pH values significantly influence the crystal growth. In acidic media (pH = 2), crystal growth has not been achieved. At pH = 4, the yield is low (10%), and the CTO presents irregular morphology. At pH = 6, the yield increases (up to 71%) obtaining an agglomeration of nanoparticles into spherical morphology. At basic conditions (pH = 8), the yield increases up to 90% and the morphology is the same as the sample obtained at pH = 6. At high basic conditions (pH = 10), the yield is similar (92%), although the morphology changes totally to dispersed nanoparticles. Importantly, the as-prepared CTO semiconductor presents photocatalytic activity for H_2_ production using triethanolamine as a sacrificial agent under visible light illumination. The results also revealed that the nanoparticles agglomerated in a spherical morphology with larger surface area presented almost double activities in H_2_ production compared to heterogeneously sized particles. These results highlight the suitable optoelectronic properties, including optical band gap, energy levels, and photoconductivity of CTO semiconductors for their use in photocatalytic H_2_ production.

## Abstract 2

Shape-controlled synthesis of Cu_3_TeO_6_ (CTO) using NH_4_OH and without any template or a calcination step was demonstrated. The modification of pH values influenced the crystal growth and morphology of the nanoparticles. The CTOs prepared in this work demonstrated photocatalytic features in H_2_ production. This work opens up the possibility of synthesizing MTOs with different morphologies to be applied in energetic applications.

## Introduction

The design and crystal growth of semiconductor materials have been drawing attention of the scientific community due to the materials’ applications in photocatalysis, photosensors, and solar cells, among others.^1−3^ A promising family of semiconductor materials is metal tellurates M_3_TeO_6_ (MTOs), which are composed of 3d transition metals (M) such as copper (Cu), nickel (Ni), cobalt (Co), tellurium (Te), and oxygen (O).^4,5^ Until now magnetoelectric applications have been the main focus for the use of MTOs, due to their multiferroic properties.^6,7^ Although, in the last years the scientific community has started to explore the application of these materials in the fields of photocatalysis,^5^ electrochemistry,^8^ and sensors,^9^ among others.^10^

One fascinating compound within the MTO family is Cu_3_TeO_6_ (CTO).^4,11^ The CTO crystal structure was first determined by Hostachy and Coing-boyat^12^ and was revised in 1978.^13^ This material has a cubic structure (Ia3), which is built up by TeO_6_ units connected through copper atoms.^13^ However, one of the main challenges of the application of MTOs for sensors, solar cells, or photocatalysis, is the synthetic procedure.^7,14^ A main synthetic route relies on the solid-state synthesis, but it fails to control the Te coordination, the morphology, and the particle size of the materials.^13,15^ Besides, this methodology requires high temperatures. The above pros and cons demand the shape-controllable synthesis of MTOs, such as wet chemical routes, to open the door to this family of materials to several applications.^9,16^ Therefore, the emphasis has been shifted toward developing CTO materials using wet chemical methodologies, such as the coprecipitation method. In this sense, Mutharani et al. showed a facile wet chemical method to prepare CTOs for their application in an electrochemical sensor.^9^ In some cases, these routes use different additives to achieve the desired composition.^4,17^ However, the use of additives such as NaOH to start the precipitation and to control the morphology of the final materials can generate different phases in the target product, e.g., Na_2_Cu_2_TeO_6_.^18,19^ In a recent study, our research group^4^ proposed the use of hydrothermal methodology to perform the synthesis of MTOs, Ni_3_TeO_6_ and Cu_3_TeO_6_, using NaOH as an additive. We obtained single-phase crystals using the hydrothermal method at pH = 7. On the other hand, the coprecipitation method at pH = 7 in the presence of NaOH leads to a multiphase in the final product. With this in mind, the design of novel synthetic strategies of MTOs, using novel procedures such as hydrothermal synthesis to control the morphology and the MTO properties, is crucial to unveil the plethora of potential applications of MTOs.

One potential application of MTOs might be their use as photocatalysts.^20,21^ Photocatalysis technology has received tremendous attention due to its promising application in renewable energy production such as H_2_ generation after the pioneer work of Fujishima and Honda about water photolysis on TiO_2_ electrodes in 1972.^22^ Recently, the scaling up of solar H_2_ production via photocatalytic overall water splitting was possible using an aluminum-doped strontium titanate particulate photocatalyst.^23^ Nevertheless, the authors claimed that it is necessary to develop photocatalysts active under visible light with adequate redox potential to boost their catalytic activity. Therefore, the scientific community is developing new materials^24,25^ or tuning widely known photocatalytic active semiconductors (TiO_2_, ZnO, etc.)^26,27^ for their application in this area. In this sense, the MTOs present great potential to be used as photocatalysts as demonstrated by their photocatalytic activity in pollutant degradation^5^ or H_2_ production.^10,28^ Additionally, these materials can absorb visible light,^11^ which represents a key point for their application in photocatalysis.

In this work, CTO nanoparticles have been synthesized by hydrothermal synthesis at different pH values using HCl and NH_4_OH to control the crystal growth and develop a shape-controlled CTO without any template or a calcination step. The as-synthesized materials have been characterized by X-ray diffraction (XRD), scanning electron microscopy (SEM), transmission electron microscopy (TEM), N_2_ adsorption, diffuse reflectance ultraviolet–visible (DRUV–vis) spectroscopy, and X-ray photoelectron spectroscopy (XPS), showing a change in morphology and yield of the final CTOs at different pH values, hydrothermal conditions such as temperature and time, and an eventual calcination step. The obtained CTOs have absorption in the visible range (*E*_g_ = 2.6 eV) and photocatalytic activity in H_2_ production, providing a preliminary proof of concept for the use of this novel material in energetic applications.

## Experimental Section

### Materials

Copper nitrate (Cu(NO_3_)_2_ × 3H_2_O, 99–104%, Sigma-Aldrich), telluric acid (H_6_O_6_Te, 98%, Sigma-Aldrich), ammonia solution (NH_4_OH, 25%, Sigma-Aldrich), absolute ethanol (EtOH, 99.5%, ETAX), and deionized (DI) water were used in the present work. All reactants were used as received without any further purification.

### Materials Preparation

CTOs were prepared by hydrothermal synthesis using Cu(NO_3_)_2_ × 3H_2_O and H_6_O_6_Te as reagents with different synthetic conditions, such as pH, hydrothermal conditions, and for comparison, a calcination step. In a typical procedure, Cu(NO_3_)_2_ × 3H_2_O and H_6_O_6_Te were mixed in a stoichiometric ratio of 3:1, respectively, in DI water (60 mL). This solution was stirred vigorously for 10 min. Then, HCl (5% v/v) or NH_4_OH (5% v/v) solution was used to adjust the pH to 2, 4, 6, 8, and 10 while stirring, to study the effect of pH in the synthetic media. The solution was adjusted to the adequate pH and was stirred for a further 5 min. Afterward, this solution was transferred to a Teflon-lined stainless-steel autoclave (100 mL) and heated to 180 °C for 12 h. After that, the mixtures were centrifuged (5000 rpm) to collect the solid materials, which were washed three times with DI water and three times with EtOH to clean the samples from impurities. The obtained solids were dried under vacuum overnight. The obtained Cu_3_TeO_6_ powders were named CTO_X, where the “X” corresponds to the pH used during the synthesis of the MTOs. The sample prepared in acidic conditions (pH = 2) was heated to 180 °C in an autoclave for 7 days. In this acidic condition, no solid semiconductor was obtained.

To study the effect of calcination, sample CTO_8 was calcined at 600 °C for 2 h with a heating rate of 10 °C/min. The calcined sample was named CTO_Calc. To study the effect of hydrothermal temperature and time, one sample was prepared using a hydrothermal temperature of 120 °C for 6 h, and another was prepared at room temperature and ambient pressure for 12 h. The Cu_3_TeO_6_ powders obtained were named CTO_120_6 and CTO_RT, respectively.

### Materials Characterization

The global yield values were calculated according to
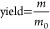
where *m* is the final mass obtained using the hydrothermal reaction and *m*_0_ is the calculated theoretical mass. Field emission SEM (FE-SEM) images were taken using a Zeiss Ultra plus field emission scanning electron microscope. The samples were coated with Pt to avoid a charging effect in the image acquisition. TEM images coupled with energy dispersive spectroscopy (EDS) mapping were obtained using JEOL JEM-2200FS field emission transmission electron microscope/scanning transmission electron microscope. Powder XRD patterns at room temperature were obtained with a Rigaku SmartLab 9 kW equipped with a five-axis θ–θ goniometer, 1D solid-state detector, and scintillator using Co–Kα (λ = 1.79 Å, 40 kV, 135 mA) radiation. The mean crystal size was estimated by applying the Scherrer equation^29^ using the full width at half-maximum (fwhm) data of the major diffraction peak and a *K* factor of 0.93
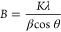
where *B* is the crystalline size (nm); *K* is the dimensionless shape factor whose value is 0.93; λ is the wavelength of the radiation source used, which is 1.79 Å for Co–Kα radiation; β is the fwhm intensity (radians), and θ is the Bragg angle at the position of the peak maximum.

The specific surface areas, average pore sizes, and volumes of the synthesized materials were measured with N_2_ adsorption at −195 °C using a Micromeritics ASAP 2020 surface analyzer. Before the analyses, the samples were evacuated for 4 h at 250 °C. DRUV–vis spectra were obtained using a Shimadzu UV-2600 spectrophotometer using BaSO_4_ as a background. The surface-sensitive technique XPS was performed with Al–Kα using a Thermo Fisher Scientific ESCALAB 250Xi XPS system. Energy calibration of the XPS was performed using C 1s peak at 284.8 eV as a reference.

The valence band edge (*E*_v_) values of the solid samples on an indium tin oxide-coated glass substrate were measured by ambient photoemission spectroscopy (APS02, KP Technology). The samples were illuminated by monochromatic UV light in the energy range of 7–4 eV and the cube root photoemission signals were extrapolated to zero to determine E_v_. The optical band gap values of the samples were added to the E_v_ to calculate the conduction band edge (*E*_c_) values. The Fermi level of the samples was recorded by a vibrating tip Kelvin probe (SKP5050, KP Technology). A freshly cleaned silver reference was used to determine the tip’s work function.

Photoelectrochemical measurements were performed in a three-electrode setup (Ivium Compacstat potentiostat) with a Ag/AgCl reference electrode and Pt counter electrode in a 0.1 M KNO_3_ aqueous electrolyte. The photoelectrodes were illuminated by a xenon lamp (Lot Quantum Design) with an AM1.5 filter and 100 mW cm^–2^ intensity through a circular mask (0.28 cm^2^). The photoelectrode was prepared by drop-casting a CTO dispersion [20 mg in 0.5 mL of ethanol and 20 μL of Nafion solution (5 wt %)] on fluorine-doped tin oxide-coated glass, which was consecutively dried at 80 °C.

### Photocatalytic Test

The photocatalytic H_2_ production of CTO synthesized at different pH values was measured under visible light irradiation on a Perfect Light PCX50B photoreactor, following the studies reported previously by our research group.^30,31^ The white LED (λ > 420 nm), employed as the irradiation source in the photoreactor used, has a light intensity of 80 mW·cm^–2^. The photocatalytic tests were performed in a quartz bottle with a height of 90 mm, a diameter of 35 mm, and a total volume of 68 mL. CTO semiconductors (5 mg) were suspended in a solution of DI water (25 mL) and triethanolamine (5%) as the electron donor. Before illumination with visible light, the dispersion was purged with Ar for 30 min under stirring conditions in the dark. The prepared solution was exposed to visible light for 4 h at room temperature using the Perfect Light PCX50B photoreactor. The H_2_ generated was measured by an Agilent 8860 gas chromatograph equipped with a split/splitless inlet, a TCD detector, and a capillary column CP-Molsieve 5 Å 25 m × 0.53 mm × 50 mm. Additionally, a blank test without catalyst was performed under the same experimental conditions as in the catalytic tests, and no catalytic activity was detected in the absence of the photocatalyst.

## Results and Discussion

### Materials Characterization

**Scheme 1:**
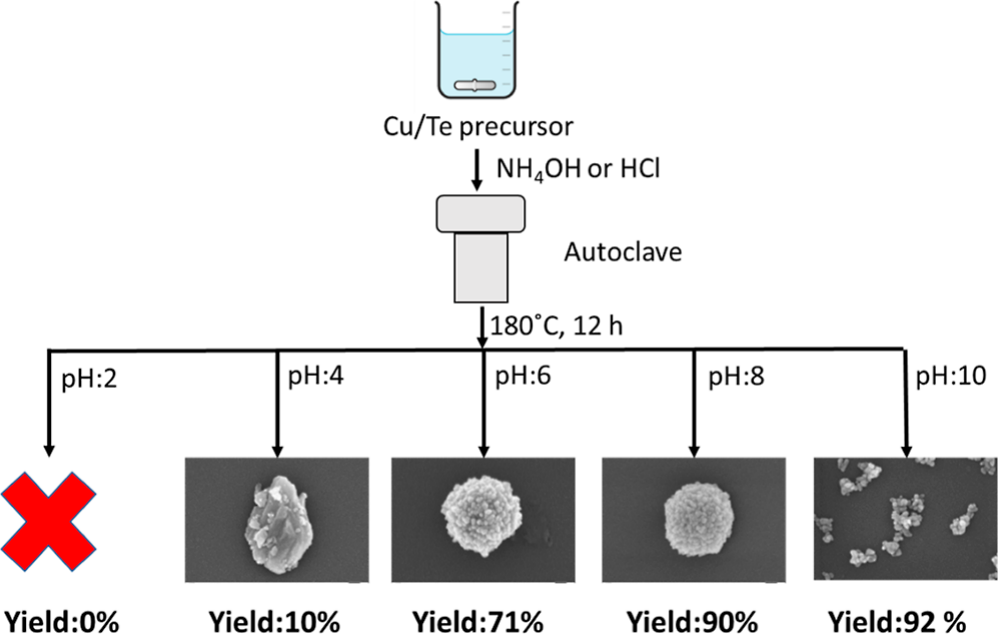
Scheme of Hydrothermal Procedure to Synthesize CTO at Different pH Values, Indicating the Morphologies and the Yields Obtained

The yields of the samples obtained at different pH values by hydrothermal synthesis without a template using HCl and NH_4_OH were calculated and are depicted in Scheme 1. Successful synthesis in acid media (pH = 2) is not observed or leads to a very low yield of 10% at pH = 4. However, at pH = 6, the obtained yield presents a great increase up to 71% and this trend is followed in basic conditions, which bring to yields of around 90 and 92% for pH = 8 and pH = 10, respectively. The results indicate that the pH value has a great effect on the synthesis of CTOs. This effect might be related to the fact that in acidic conditions copper was dissolved in an aqueous environment as Cu^2+^ ions, and with the increase of the pH, copper started to precipitate in the form of Cu(OH)_2_ as suggested by the Pourbaix diagram described by Celante and Freitas.^32^ The results suggest that for the synthesis of CTO, it is recommendable to use neutral or basic pH conditions to start the precipitation of copper with the further possibility to obtain Cu_3_TeO_6_. The growth of this crystal is enabled by the coordination of Cu(OH)_2_ with Te. On the other hand, in acidic media, copper cannot be stabilized in a crystal structure and is kept dissolved as Cu^2+^ as suggested by the Pourbaix diagram; thus, the crystal growth is not possible. Herein, we demonstrated the relevance of the pH value for the synthesis of semiconductors using wet chemistry methodologies as observed in previous reports.^32−34^

**Figure 1:**
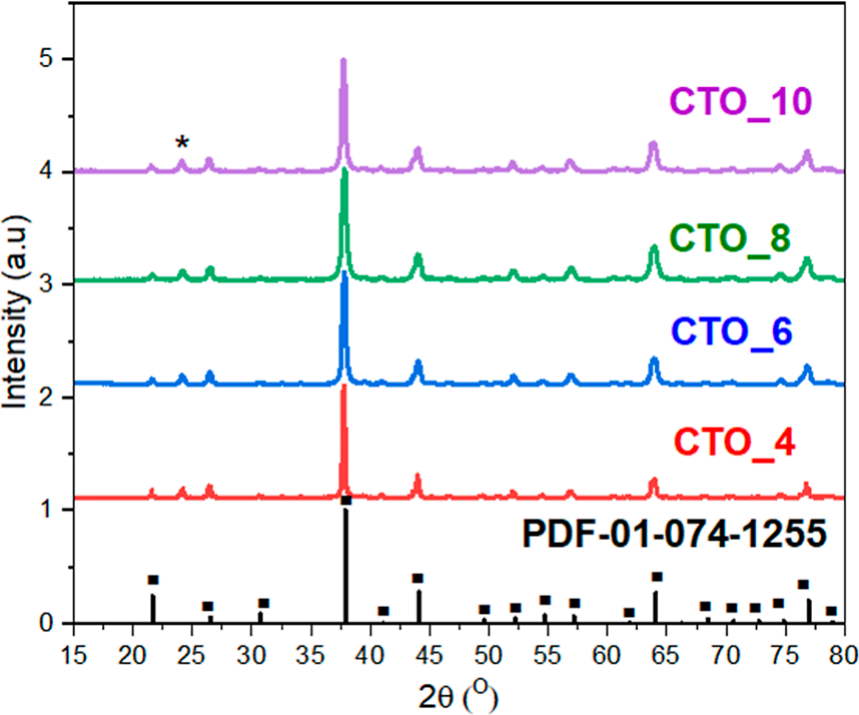
XRD patterns of CTOs synthesized by using different pH values. Square-ended bars indicate the position and relative intensity of Cu_3_TeO_6_ reflections (PDF-01-074-1255). (*) indicates the position of Cu_3_TeO_6_·H_2_O reflection (PDF-00-047-1751).

The crystal structures of the CTOs synthesized at different pH values were studied by XRD analysis (Figure 1). Independently from the pH synthesis media, all the samples prepared in this work showed the characteristic diffraction peaks at 2θ = 20.93, 25.90, 30.10, 37,25, 43.42, 54.07, 56.52, 63.48, 70.05, and 76.377°, corresponding to the characteristic peaks of (200), (211), (220), (222), (400), (422), (431), (440), (600), and (622) lattice planes of Cu_3_TeO_6_ (PDF 01-074-1255), respectively, and related to Cu_3_TeO_6_ crystalline single phase.^35^ However, the samples also show a characteristic diffraction peak at 2θ = 24.24° corresponding to the characteristic peak of (210) of mineral mcalpineite “Cu_3_TeO_6_ × H_2_O”. This fact indicates that the CTO synthesized by hydrothermal methodology presents both phases due to the aqueous environment which allows for the incorporation of water molecules in the crystal structure of Cu_3_TeO_6_ as previously reported in the literature.^36^ Moreover, the crystallite sizes of the MTOs synthesized at different pH values were calculated using the Scherrer equation. The results indicate that upon acidic conditions (pH = 4) a bigger crystallite grain size (76.2 nm) was formed while the CTO synthesized at higher (basic) pH such as pH = 6, pH = 8, and pH = 10 had crystallite grain sizes of 30.1 26.4, and 29.7 nm, respectively. These calculations indicate that the pH of the reaction media has a great influence on the crystalline size as it will be further displayed with microscopic analysis results.

**Figure 2:**
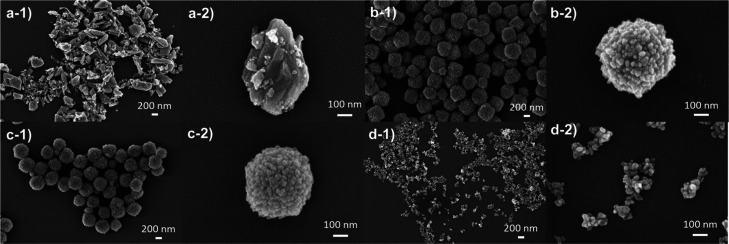
SEM micrographs of CTOs prepared at different pH values: (a) 4, (b) 6, (c) 8, and (d) 10.

The morphology of the samples prepared by hydrothermal synthesis using different pH values (CTO_4, CTO_6, CTO_8, and CTO_10) have been studied by SEM and TEM. The SEM micrographs (Figure 2) show that the pH of hydrothermal synthesis had a great effect on the morphology of the materials as previously reported for several other semiconductors.^37−39^ The sample synthesized at acidic pH (pH = 4) had an irregular size (160 nm). The morphology in Figures 2a and S1a demonstrates heterogeneous size and shape, with an average size of 160 nm (Figure S1a). However, at pH = 6 or 8, the observed materials revealed a homogeneously dispersed size and spherical morphology due to the agglomeration of smaller nanoparticles in these specific synthetic conditions as shown in Figure 2b,c. The sample CTO_6 is formed by nanoparticles with a smaller size (24 nm) (Figure S1b) and aggregated to form spherical particles with an average size of 388 nm (Figure S2a). The same effect was observed for the CTO_8 sample, which exhibited a slight increase in the size of the nanoparticles (26 nm) (Figure S1c) and in the size of the spherical agglomerations (390 nm) (Figure S2b) with respect to the CTO_6 sample. At basic synthetic conditions (pH = 10), the sample CTO_10 presents a homogeneously dispersed nanoparticle size (28 nm) (Figure S1d) without aggregation in spherical morphology (see Figure 2d). To confirm the result obtained by SEM, the samples were also analyzed by TEM (Figure S3). TEM analysis was consistent with the results obtained by SEM since sample CTO_4 presents an irregular morphology. Furthermore, the samples CTO_6 and CTO_8 have a spherical morphology due to the agglomeration of small nanoparticles as was observed by SEM analysis. Also, it is observed that sample CTO_10 possesses a nanoparticle morphology without agglomeration in spherical particles, contrary to CTO_6 and CTO_8. The SEM and TEM results indicated that the pH condition in the hydrothermal synthesis of MTOs substantially affects the morphology, size, and crystallinity of the tellurates. Moreover, SEM and TEM images show that at pH = 6 and pH = 8 the nanoparticles tend to aggregate to decrease the surface energy. Indeed, it is well-known and has been already reported that the surface energy is minimal on spheric-shaped morphologies.^38,40^

**Figure 3:**
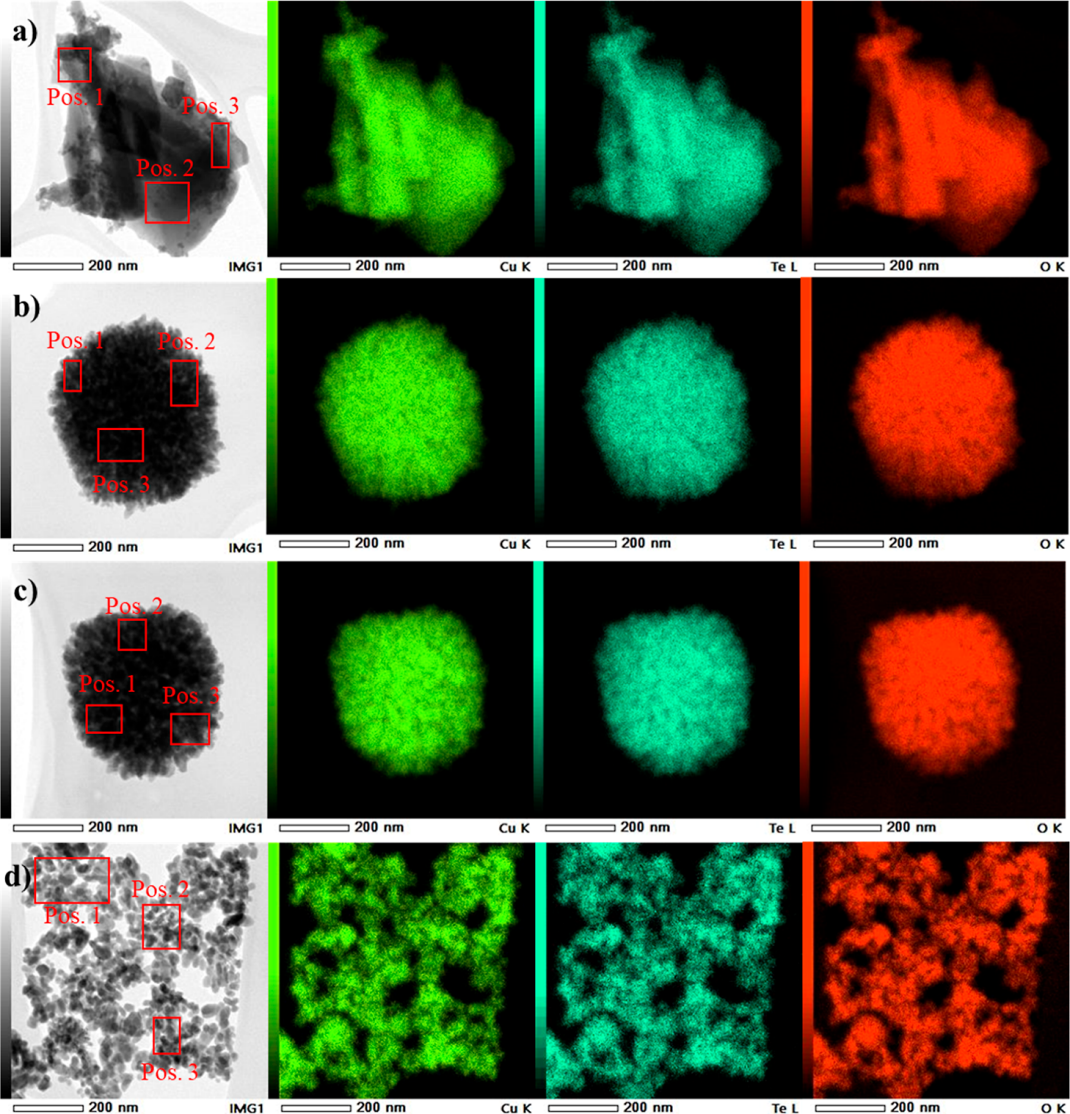
STEM images and corresponding EDS mapping images of Cu (green), Te (blue), and O (red) elements. (a) STEM image and elemental mapping images of CTO_4. (b) STEM image and elemental mapping images of CTO_6. (c) STEM image and elemental mapping images of CTO_8. (d) STEM image and elemental mapping images of CTO_10.

The elemental composition of the CTO samples (Figure 1g) was analyzed by STEM-EDS. The STEM images (Figure 3) show the same morphology dependent on the pH media used as was observed by SEM and TEM analyses. The EDS mappings (Figure 3) of the samples show only the presence of Cu (green), Te (blue), and O (red) elements homogeneously distributed in the sample, regardless of the morphology obtained. The relative amount obtained by EDS of the elements Cu and Te is lower (Table S1) with respect to O compared with the stoichiometric ratio (Cu_3_TeO_6_) and our previously reported results.^4^ This fact might indicate the presence of both phases (Cu_3_TeO_6_ and Cu_3_TeO_6_·H_2_O) in the samples synthesized in this work as it was observed by XRD analysis, since the Cu_3_TeO_6_·H_2_O material has a higher stoichiometrical amount of O in its elemental structure.

**Figure 4:**
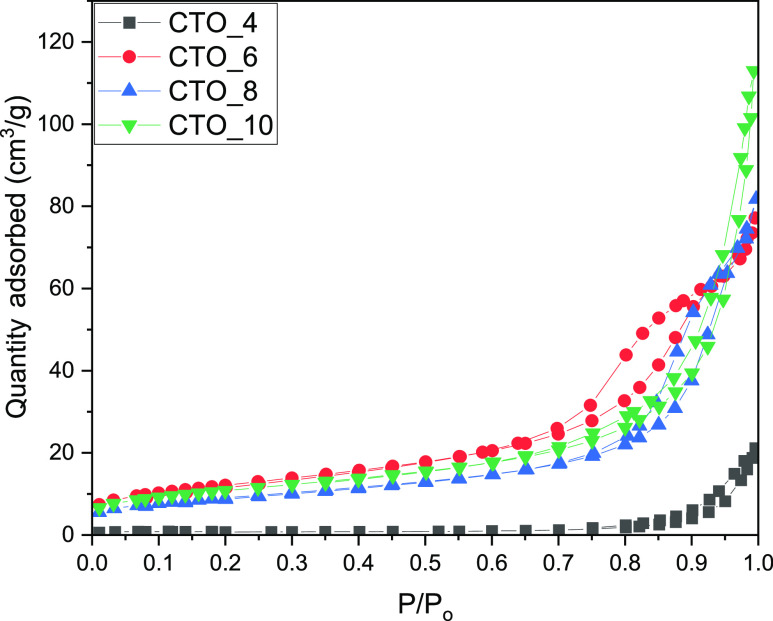
N_2_ isotherms at 77 K for the samples prepared in this study at different pH values.

The porous texture of the synthesized samples was investigated by N_2_ adsorption measurements (Figure 4). CTO_4 sample synthesized at acidic conditions revealed a type II isotherm, typical of nonporous materials.^41^ The CTO_6 sample had a type IV isotherm, indicating that the structure is mesoporous due to the agglomeration of nanoparticles in spherical morphology as observed by SEM and TEM analyses. Moreover, the CTO_8 sample showed a type IV isotherm indicating that the material is mesoporous.^42,43^ However, the hysteresis of the CTO_8 samples was significantly smaller than sample CTO_6 implying a change in the mesoporosity of this sample.^42^ The CTO_10 sample had a type IV isotherm although the hysteresis decreased. Indeed, at these synthetic conditions, the sample presents a nanoparticle morphology without aggregation of nanoparticles in spheres as it was previously described by SEM.^43^ Concerning the textural properties shown in Table 1, the changes in the synthetic conditions, focusing on the pH, have a great impact on the surface area of the materials. The CTO_4 sample synthesized in acidic media had a low surface area indicating that this material is nonporous with big particle sizes as was observed by N_2_ adsorption isotherms and SEM analysis. The samples synthesized at higher pH (6, 8, and 10) demonstrated an increase in the surface area. The CTO_6 sample showed a higher surface area (43.1 m^2^/g) due to the small nanoparticles observed by SEM and the space generated between the particles in the spherical agglomerations.^44^ However, the increase in the pH value, in the samples CTO_8 and CTO_10, corresponds to a decrease in the surface area to 36.1 and 36 m^2^/g, respectively, probably due to the loss of the spherical features and to the change in the mesoporosity as indicated by the change in the hysteresis of the isotherms (Figure 4).

**Table 1:** Textural Properties of CTOs Prepared at Different pH Values

samples	*S*_BET_ (m^2^/g)	*V*_total,0.95_ (cm^3^/g)	*V*_N2DR_ (cm^3^/g)
CTO_4	2.2	0.025	0.0014
CTO_6	43	0.115	0.0316
CTO_8	36	0.112	0.029
CTO_10	36	0.167	0.0257

**Figure 5:**
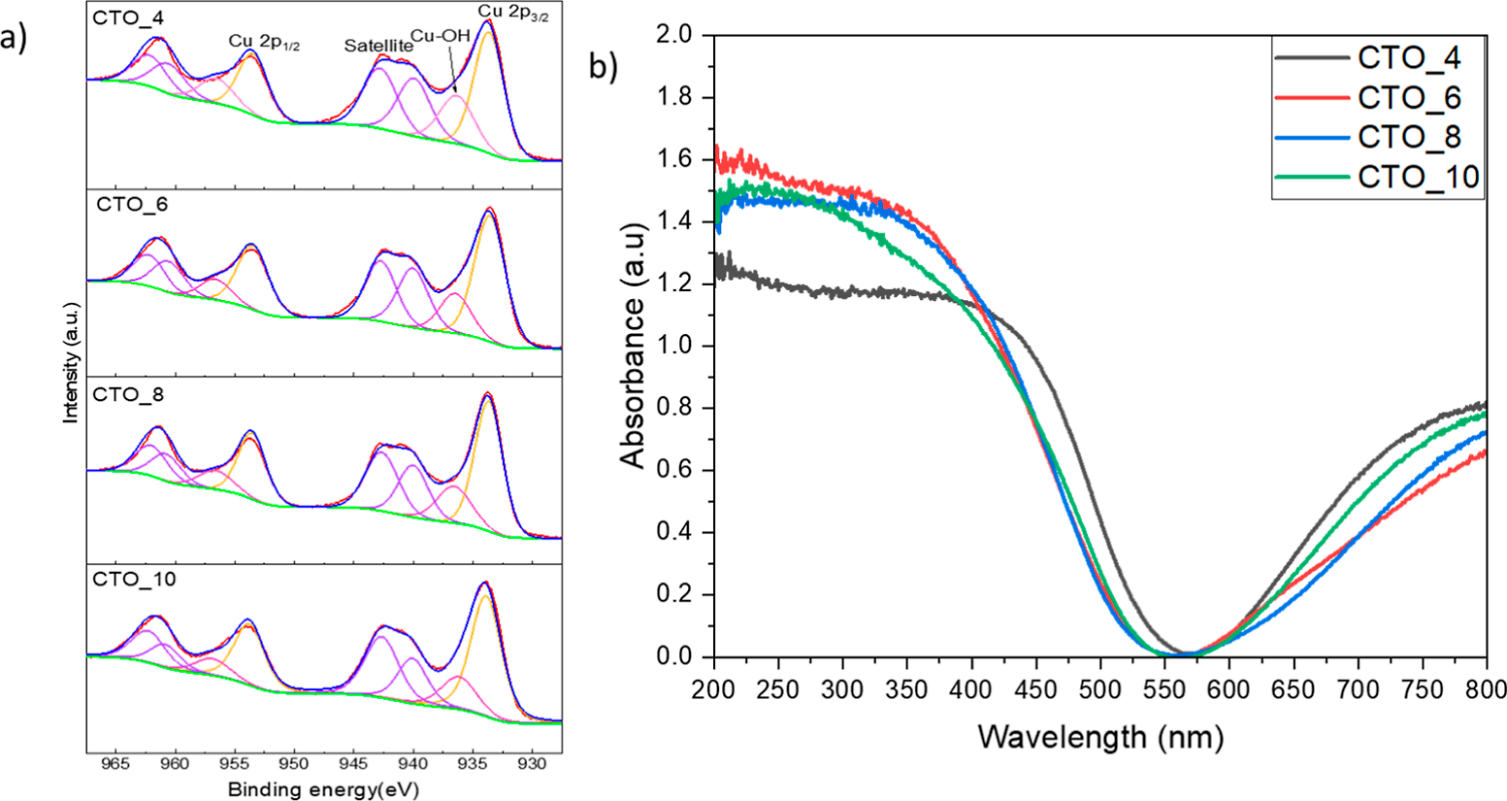
(a) XPS spectra of Cu 2p and (b) DRUV–vis spectra of samples prepared in this work at different pH values.

The chemical composition and the oxidation states of the elements in the prepared samples and the optical absorption properties were analyzed by XPS and DRUV–vis analysis, respectively. The XPS analysis of Cu 2p level (Figure 5a) for the samples prepared at different pH shows a peak at 933.7 eV and a satellite peak at 940.1 eV for CTO_4, CTO_6, CTO_8, and CTO_10, indicating the presence of Cu^2+^.^11^ Also, in all the samples prepared using hydrothermal synthesis a peak at 936.66 eV is observed, indicating the presence of Cu(OH)_2_^45^ on the surface of the material due to the use of wet chemistry conditions, more specifically of aqueous environment. The presence of Cu(OH)_2_ in the CTO samples prepared by hydrothermal synthesis was observed and described previously in our recent work.^4^ In this work, the analysis of Te 3d XPS spectra for the CTO samples was performed (Figure S4a), despite the fact that the analysis is challenging due to the Cu-auger peak appearing at the same range (569.2 eV). To solve this problem, the Cu-auger peak was fitted to obtain the correct background and to successfully perform the analysis of Te 3d XPS peaks. Te 3d XPS spectra showed two peaks at 575.8 and 576.9 eV, indicating the presence of Te^4+^ and Te^6+^, respectively. The mix valence between Te^4+^ and Te^6+^ was recently reported by Numan et al. for Ni_3_TeO_6_^46,47^ and observed in our recent report.^4^ The O 1s XPS spectra of CTO samples showed a main peak at 530.46 eV assigned to the oxygen present in Cu_3_TeO_6_ lattices. Additionally, a shoulder at 531.3 eV indicates the possible presence of –OH on the surface of CTO samples or oxygen vacancies in agreement with Cu 2p XPS analysis and with the previous results reported by our group^4^ and the group of Numan et al.^46,47^ Finally, the results of XPS for the CTO materials synthesized at different pH values revealed the presence of Cu^2+^, Te^4+^ and Te^6+^, which might be generated by the possible oxygen vacancies.^46,47^ Moreover, the presence of –OH functional groups on the surface of the samples, even at pH = 4, indicates that the Cu(OH)_2_ intermediate might be crucial for the synthesis of CTOs as was observed following the yield of the synthesis.

The DRUV–vis spectra of the samples prepared at different pH values are shown in Figure 5b. All CTO samples showed two absorption ranges and an abrupt cutoff in the range of 200–500 nm as common in semiconductors.^4,11^ The band gaps (*E*_g_) determined by Tauc plot analysis^48^ (Figure S5) demonstrate that the sample synthesized at acidic pH (CTO_4) had a lower band gap (2.46 eV) than the samples prepared at higher pH (CTO_6:2.6 eV, CTO_8:2.59 eV, and CTO_10:2.53 eV). The DRUV–vis results indicated that CTO is a semiconductor with a band gap in the range of the visible (2.6 eV), which is a very intriguing property for its use as a photocatalyst in visible light-mediated processes.^5,20,49^

Based on the above results, a possible scheme of the formation mechanism of the Cu_3_TeO_6_ materials prepared in this work using hydrothermal synthesis might be expressed as follows
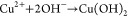
1
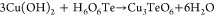
2

To start the growth of the crystals, the copper ions present in the solution are likely to be coordinated with the ion hydroxide to form copper hydroxide (Cu(OH)_2_). Then, copper hydroxide reacts with tellurium precursor (H_6_O_6_Te). The temperature and the pressure achieved in the hydrothermal conditions lead to the formation of crystals of copper tellurate (Cu_3_TeO_6_), following the mechanism previously reported for several inorganic materials using hydrothermal synthesis.^50,51^ This mechanism is proposed since in acidic media (pH = 2) the synthesis of MTOs is not achieved even after performing the reaction for 1 week. At pH = 4, the material is synthesized but the yield of the reaction is very low (10%) and also in this case the functional group –OH and extra phase Cu_3_TeO_6_·H_2_O were observed by XPS and XRD analysis, respectively. When a base (NH_4_OH) was used in the solution, the yield of the reaction increased to 71% (CTO_6), 90% (CTO_8), and 92% (CTO_10). This fact indicates the great effect of OH^–^ ions in the synthesis of MTOs. Another proof supporting this possible mechanism is that the Pourbaix diagram described by Celante and Freitas^32^ indicates that at pH higher than ∼6 the Cu^2+^ starts the coordination and later precipitates in the form of Cu(OH)_2_ in agreement with our observations. This points toward the importance of Cu(OH)_2_ as a possible intermediate in the formation of CTO.

**Figure 6:**
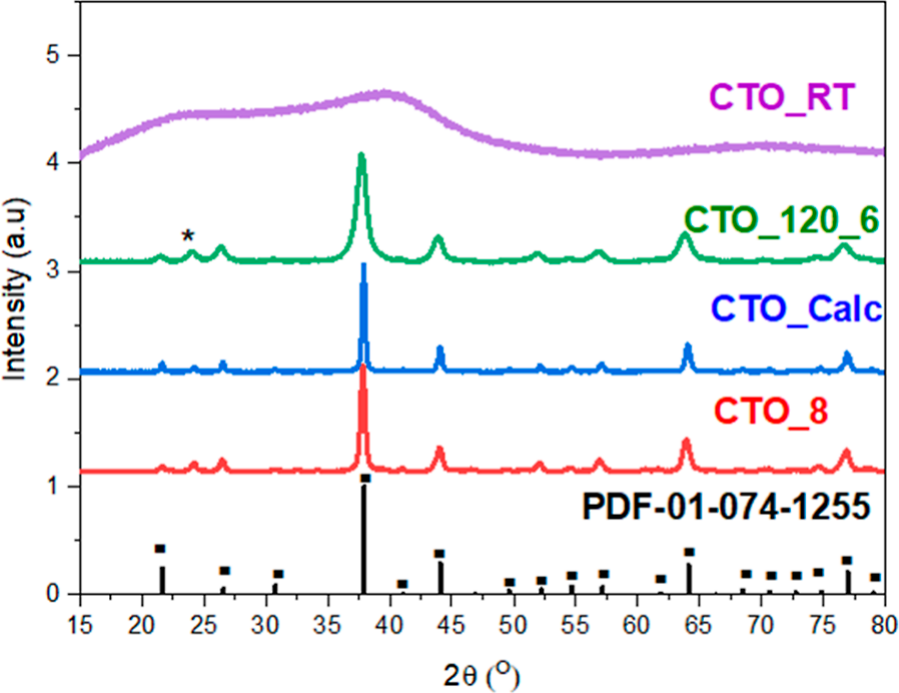
XRD patterns of CTO materials using different synthetic conditions (hydrothermal and calcination temperature). Square-ended bars indicate the position and relative intensity of Cu_3_TeO_6_ reflections (PDF-01-074-1255). (*) indicates the position of Cu_3_TeO_6_·H_2_O reflection (PDF-00-047-1751).

In this work, the effects of hydrothermal methodology (temperature and time) and the calcination effect on CTO materials were studied. To study the effect of the calcination step, sample CTO_8 was calcined at 600 °C (CTO_Calc). Furthermore, the effect of hydrothermal conditions such as temperature and time was studied, using a hydrothermal temperature of 120 °C for 6 h (CTO_120_6) or room temperature and ambient pressure for 12 h (CTO_RT) to study the effect of the hydrothermal synthesis (see the Experimental Section). The powder XRD analysis of the samples prepared with different conditions demonstrated different crystalline properties as shown in Figure 6. The sample calcined at 600 °C had the same crystalline phases as the sample without calcination (CTO_8). However, the intensity of the diffraction peak at 2θ = 24.24°, which corresponds to the characteristic peak of (210) of mineral mcalpineite “Cu_3_TeO_6_·H_2_O”, decreases with respect to sample CTO_8. This fact could be explained by the possible removal of H_2_O from the crystalline structure or by the elimination of the –OH functional groups present on the surface of the CTO_8 sample during the calcination step. Additionally, after the calcination step the crystalline grain of the sample increased from 26.4 (CTO_8) to 40.1 nm (CTO_8_Calc). The treatment with high temperature led to an enlargement of the particles as previously observed in several semiconductors, such as TiO_2_.^44,52^ Regarding the effect of the hydrothermal treatment, it is observed that the sample CTO_120_6 presents the same crystalline phase as CTO_8, indicating that the crystalline phase of Cu_3_TeO_6_ can be obtained at a lower hydrothermal temperature in only 6 h without the calcination step. However, the crystalline size of this sample is 10.8 nm, which indicates that the increase in temperature and time leads to an increase in the size and in the crystallinity of the MTOs as previously reported for other inorganic materials.^53^ The XRD analysis of sample CTO_RT prepared at room temperature and pressure shows that this sample is amorphous. This fact highlights the importance of hydrothermal synthesis to obtain crystalline CTOs.

**Figure 7:**
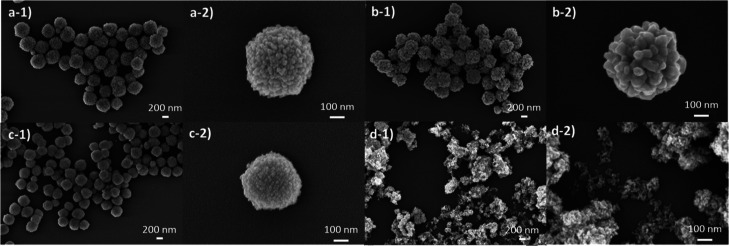
SEM micrographs of CTOs prepared at different conditions (hydrothermal and calcination step): (a) CTO_8, (b) CTO_8_Calc, (c) CTO_120_6, and (d) CTO_RT.

The morphology of the CTO_8_Calc, CTO_ 120_6, and CTO_RT samples has been studied by SEM (Figure 7) and compared with that of CTO_8 (Figure 7a). The SEM images of the sample CTO_8_Calc (Figure 7b) show that the calcination step increases the nanoparticles size from 26 (Figure S1c) to 64 nm (Figure S6a) as was observed by XRD, maintaining the spherical size due to the agglomeration of the particles. The CTO_120_6 sample (Figures 7c and S6b) has a smaller size (11 nm) than CTO_8 (26 nm), confirming the great effect of hydrothermal conditions detected also by XRD analysis. The sample prepared at room pressure and temperature (CTO_RT) shows a heterogeneous morphology, indicating that the use of the hydrothermal methodology is inevitable for shape control of the morphology. Indeed, the high temperature and pressure obtained in the autoclave when using this methodology^51,54^ allow the formation of crystals with well-defined shapes and morphology. The calcination effect on the porous texture of the Cu_3_TeO_6_ samples was determined by N_2_ adsorption measurements (Figure S7 and Table S2). As shown in Figure S7, the sample calcined at 600 °C shows a sintering effect and, as a consequence, a loss of porosity, validating the XRD and SEM results. Regarding the textural properties of the calcined sample, this sample presents a low surface area due to the sintering effect of the nanoparticles as observed in the literature for TiO_2_.^44^ The structural analysis of CTO using different synthetic parameters as the calcination step, different times and temperatures in hydrothermal synthesis, and synthesis at room temperature and pressure indicates that these parameters dramatically affect the synthesis of MTOs. In this sense, the results show that a calcination step was not necessary to obtain the crystalline MTOs. However, considering that at room temperature and pressure the materials obtained were amorphous, the use of hydrothermal synthesis is mandatory to reach crystalline MTOs due to the high pressures and temperatures generated in the autoclaves. Moreover, in this study, the significance of the hydrothermal parameters was underlined. An increase in the time and in the temperature of the hydrothermal treatment enhances crystallinity and size of the nanoparticles; hence, the hydrothermal conditions affect the final structural properties of CTO material.

**Figure 8:**
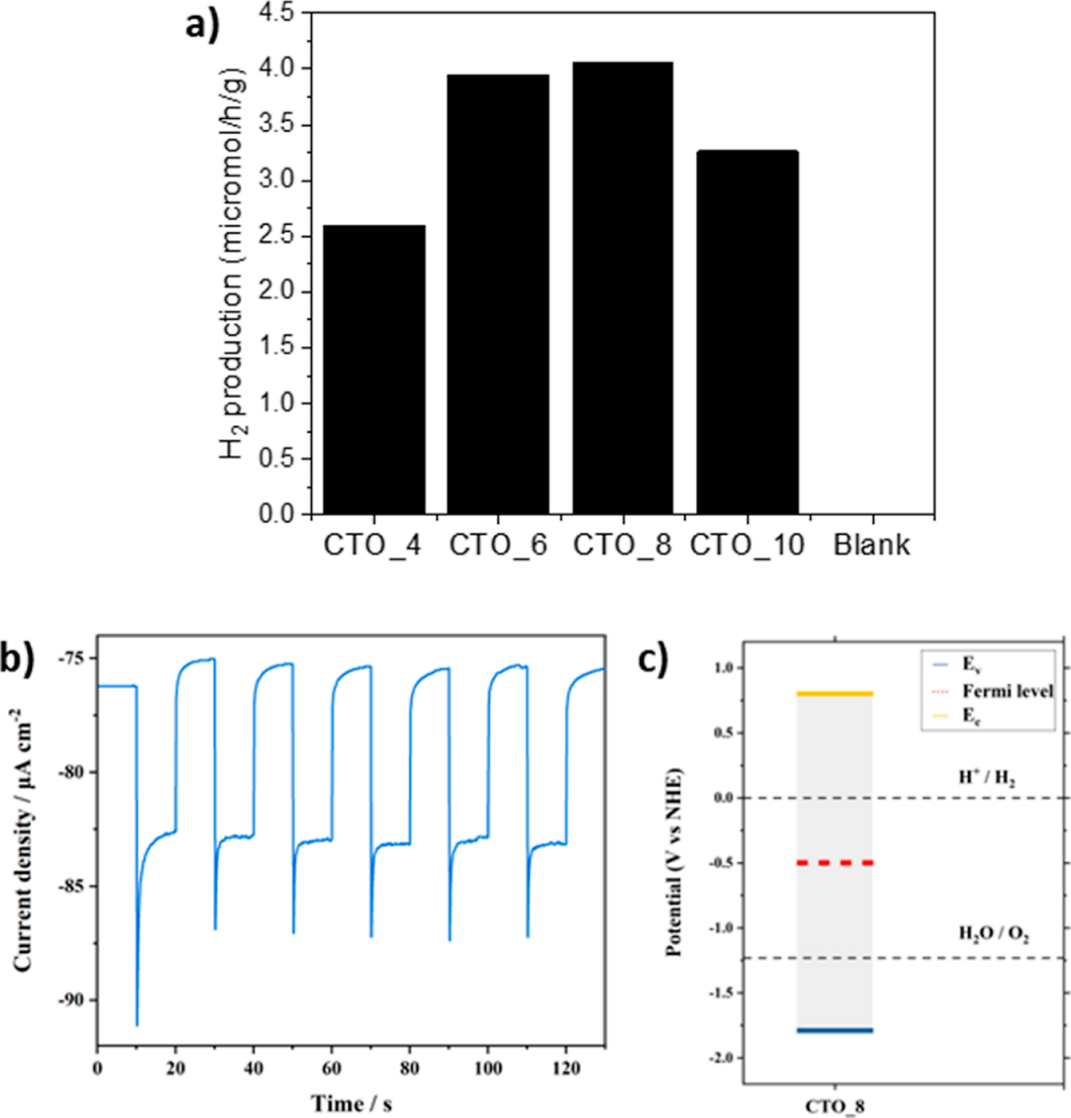
(a) H_2_ production of CTO samples prepared at different pH values and blank test without a catalyst (blank). (b) Photocurrent generated by a CTO_8 photoelectrode under chopped 1 sun illumination in an aqueous 0.1 M KNO_3_ electrolyte at 0 V vs the reversible hydrogen electrode. (c) Comparison of the measured energy levels of CTO_8 to the electrochemical potential of water oxidation and reduction.

The effect of the copper precursor (CuNO_3_, CuCl_2_, and CuSO_4_) in the hydrothermal synthesis of CTO semiconductors was also studied. The results obtained by XRD and SEM show that the copper precursor does not change the crystalline phase (Figure S8) and the morphology (Figure S9) of the synthesized CTO. This fact indicates that the Cu precursor does not have a relevant effect on the hydrothermal synthesis of the MTOs.

### Photocatalytic H_2_ Production Activity of CTO Samples

The CTO semiconductors synthesized at different pH values absorb visible light, have large surface areas, and have high surface groups on the surface. Thus, CTO semiconductors are employed in photocatalytic water splitting. The catalytic tests showed that the CTOs synthesized in this work are capable of producing H_2_ using triethanolamine (5% v/v) as an electron donor under visible light irradiation (see Figure 8a). Furthermore, the results (Figure 8a) show that the pH in the hydrothermal synthesis affects the photocatalytic activity: CTO_6 and CTO_8 show better catalytic activity than CTO_4 and CTO_10. This fact might be related to the spherical morphology obtained for the samples synthesized at pH = 6 and pH = 8. Consequently, the morphology based on nanoparticles agglomerated in spheres (CTO_6 and CTO_8) provides a mesoporous catalyst and a small particle size of CTOs to boost the H_2_ production as previously reported for other photocatalysts.^55^ These results highlight the importance of the surface area in photocatalysis since the nonporous CTO_4 sample with 2.2 m^2^/g of BET area does not present high amount of produced H_2_. On the other hand, CTO_6 (43 m^2^/g of BET area) and CTO_8 (36 m^2^/g of BET area) samples showed improved photocatalytic results. These results also underline another relevant feature of the materials presented in this work, i.e., the porosity. To an increase of the mesoporosity corresponds an easier diffusion of the reagents, and consequently, an enhanced catalytic activity as it was described previously in the literature.^56^ Therefore, this would explain the boosted catalytic activity in CTO_6 and CTO_8 (spherical morphology) samples with respect to CTO_10 (nanoparticle morphology) and CTO_4 (irregular morphology). In other words, the presence of mesoporosity in CTO_6 and CTO_8 generated by the space between the particles in the spherical agglomerations, as was observed by N_2_ adsorption and SEM analysis, results in a higher H_2_ production. Furthermore, in the photocatalytic tests, it is noticeable that the sample synthesized at pH = 10 presents higher activity than the sample synthesized at pH = 4. This is in line with morphologic determinations in SEM (Figure 2) and textural properties obtained by N_2_ adsorption analysis (Table 1) where the larger surface areas provide more reaction sites for photocatalysis. The photocatalytic activity of CTO was further confirmed by observing significant (∼17 μA/cm^2^) photocurrent generation by CTO photoelectrodes in an aqueous electrolyte at 0 V vs the reversible hydrogen electrode (see Figure 8b). Finally, the energy band diagram of CTO_8 was built based on APS and Kelvin probe measurements (see Figure 8c). The *E*_v_ value of CTO_8 at −1.79 V vs the normal hydrogen electrode is below the water oxidation potential, while the *E*_c_ at 0.8 V vs the normal hydrogen electrode is above the hydrogen evolution reaction potential with a Fermi level in the middle of the band gap. Such energy levels are desirable for photocatalysts used for solar water splitting and confirm the suitability of the CTO for photocatalytic H_2_ production. Overall, these results provide a preliminary proof of concept for the use of CTO for future energetic applications, such as photocatalytic H_2_ production.

## Conclusions

In conclusion, shape-controlled hydrothermal synthesis of Cu_3_TeO_6_ using NH_4_OH as the alternative base to NaOH was demonstrated without a calcination step. The hydrothermal synthesis conditions have a great effect on the final properties of the CTOs. In this sense, the use of basic pH to synthesize CTO materials with high crystallinity and high yield is evidenced as shown by experiments carried out at different pH values in the hydrothermal synthesis of CTO. Importantly, XRD and XPS results unveiled that Cu(OH)_2_ is a possible intermediate in the hydrothermal synthesis of CTOs.

The morphology and size of the CTO nanoparticles were observed and analyzed by SEM and XRD and could be tuned not only by modifying the synthetic conditions such as pH but also by changing the hydrothermal conditions (temperature and time) or adding a calcination step. However, to obtain crystalline MTOs at low temperatures, a hydrothermal synthesis procedure is necessary considering that an amorphous material was obtained when the synthesis was performed at room temperature and pressure.

The CTOs fabricated in this work using hydrothermal synthesis demonstrated photocatalytic activity in H_2_ production in the presence of triethanolamine as an electron donor. The photocatalytic tests showed that the morphology of the synthesized nanostructures affects the catalytic activity. Samples synthesized at pH = 6 and pH = 8 showed spherical morphology and the highest photocatalytic activity. This work opens up the possibility of synthesizing MTOs with different morphologies to be applied in energetic applications, such as photocatalysis and photoelectrochemical devices.

## Supporting Information Available

Size distribution histogram of CTO samples, TEM images, EDS spectroscopy analysis, XPS spectra of Te 3d and O 1s, Tauc plot of CTO samples, N_2_ isotherms at 77 K and textural properties, and XRD patterns and SEM images of CTO samples prepared with different precursors (PDF)
