# Supplementary material for: Shape-Controlled Synthesis of Cu3TeO6 Nanoparticles with Photocatalytic Features
Source: Cryst Growth Des. 2023 Nov 7;23(12):8828–37. doi: 10.1021/acs.cgd.3c00929 (PMC10704480; doi:10.1021/acs.cgd.3c00929)
Supplement: Supplementary file 1 — cg3c00929_si_001.pdf [file cg3c00929_si_001.pdf]

Supplementary Information

Shape-controlled synthesis of Cu_3TeO_6 nanoparticles with photocatalytic features

Javier Fernández-Catalá[1,2]*, Laura Jussila[1], Matyas Daboczi[3], Filipp Temerov[1], Salvador Eslava[3], Rossella Greco[1] and Wei Cao[1]

[1]Nano and Molecular Systems Research Unit, University of Oulu, Oulu FIN-90014, Finland.

[2] Materials Science Institute and Inorganic Chemistry Department, University of Alicante, Ap. 99, E-03080 Alicante, Spain.

[3] Department of Chemical Engineering and Centre for Processable Electronics, Imperial College London, London SW7 2AZ, U.K.

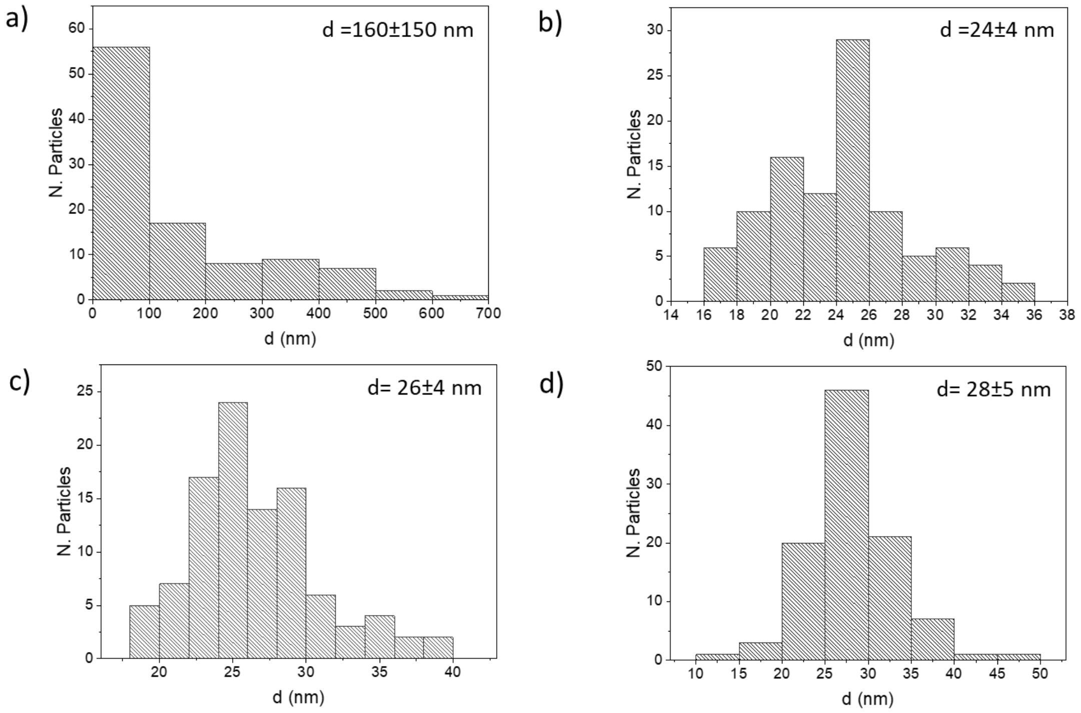

Figure S1. *Size distribution of 100 nanoparticles prepared at different pH values: a) CTO_4, b) CTO_6, c) CTO_8, and d) CTO_10.*

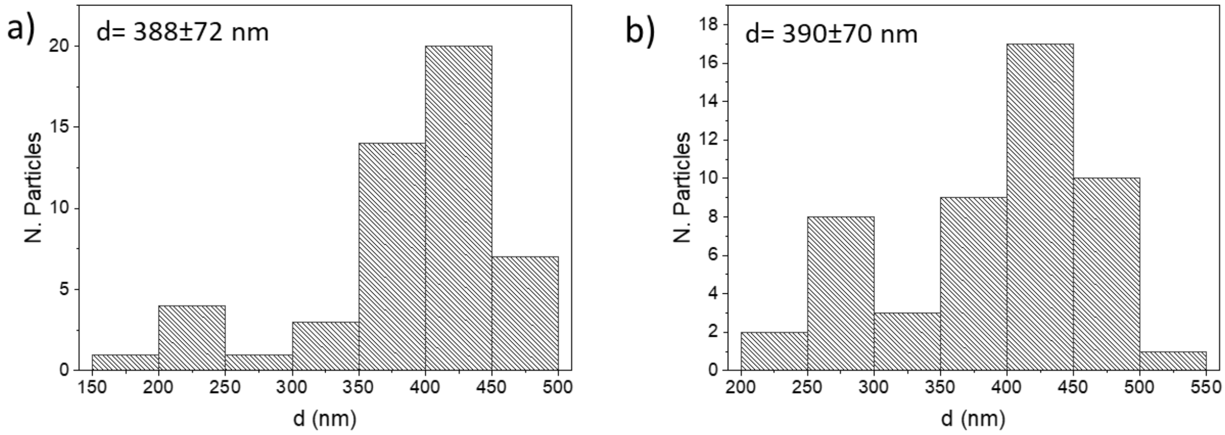

Figure S2. *Size distribution of 50 spherical-shaped nanoparticle agglomerates of a) CTO_6, and b) CTO_8.*

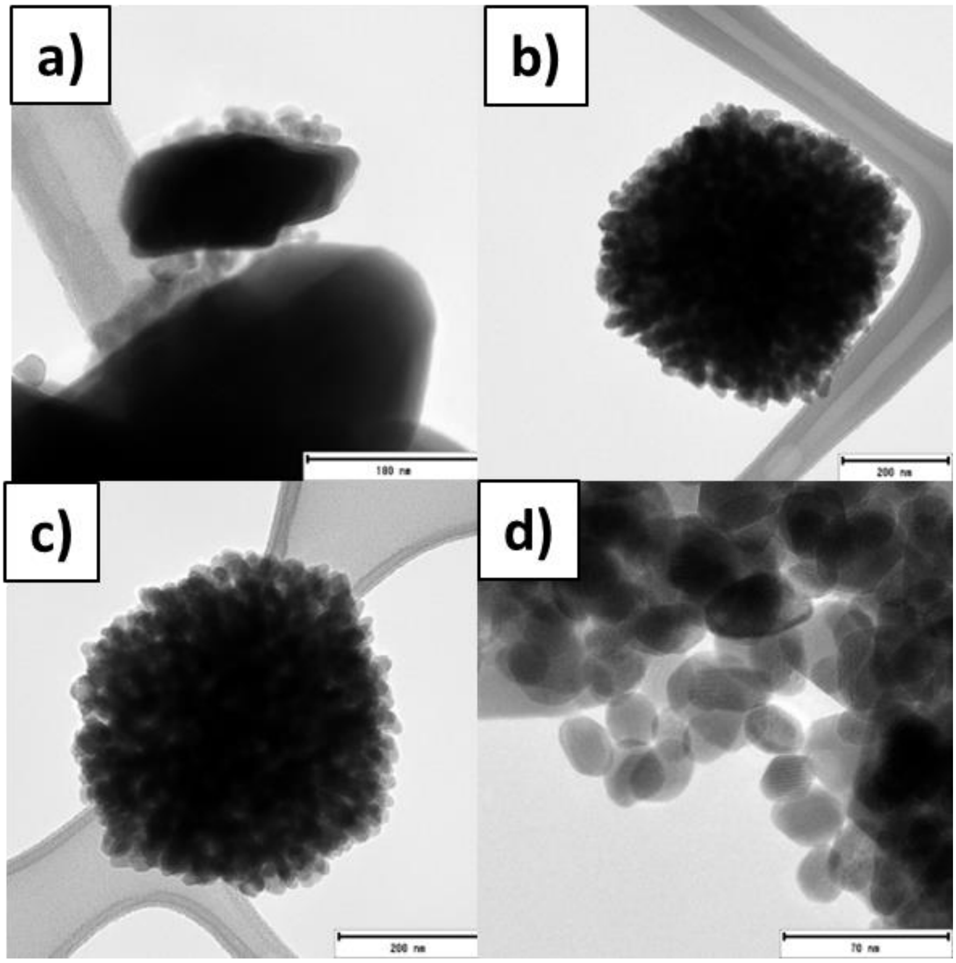

Figure S3. *TEM micrographs of CTO prepared at different pH values: a) CTO_4, b) CTO_6, c) CTO_8, and d) CTO_10.*

Table S1. *Atomic percentages of the different elements composing the materials prepared in this work obtained by EDS spectroscopy.*

Sample	***Cu (Atomic %)***	***Te (Atomic %)***	***O (Atomic %)***
CTO_4 Pos .1	*22.62*	*9.29*	*68.09*
CTO_4 Pos .2	*24.66*	*9.86*	*65.78*
CTO_4 Pos .3	*22.35*	*9.02*	*68.62*
CTO_6 Pos .1	*24.70*	*8.64*	*6.66*
CTO_6 Pos .2	*24.81*	*8.64*	*66.55*
CTO_6 Pos .3	*29.57*	*10.21*	*60.21*
CTO_8 Pos .1	*23.51*	*9.12*	*67.37*
CTO_8 Pos .2	*21.22*	*8.28*	*70.50*
CTO_8 Pos .3	*23.38*	*9.16*	*67.45*
CTO_10 Pos .1	*21.57*	*8.16*	*69.82*
CTO_10 Pos .2	*21.80*	*8.61*	*69.58*
CTO_10 Pos .3	*22.45*	*8.98*	*68.58*

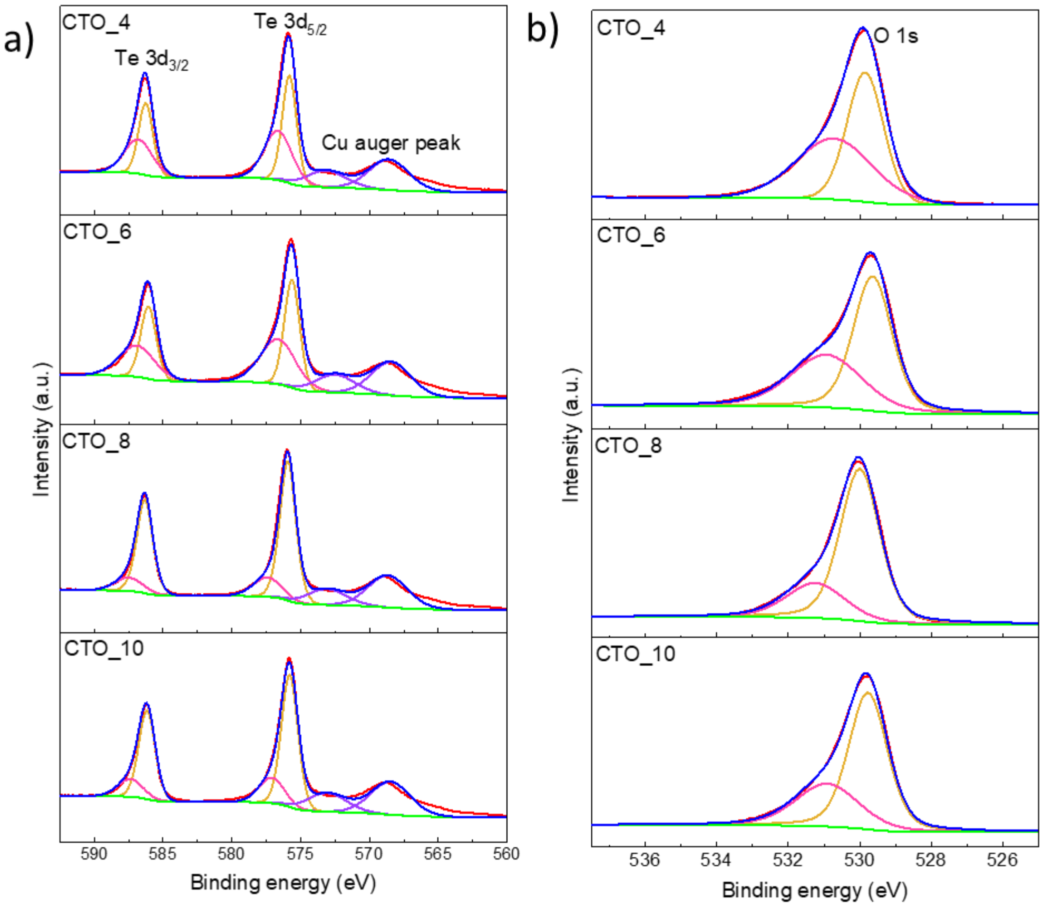

Figure S4. *XPS spectra of a) Te 3d and b) O 1s of CTO samples prepared in this work at different pH values.*

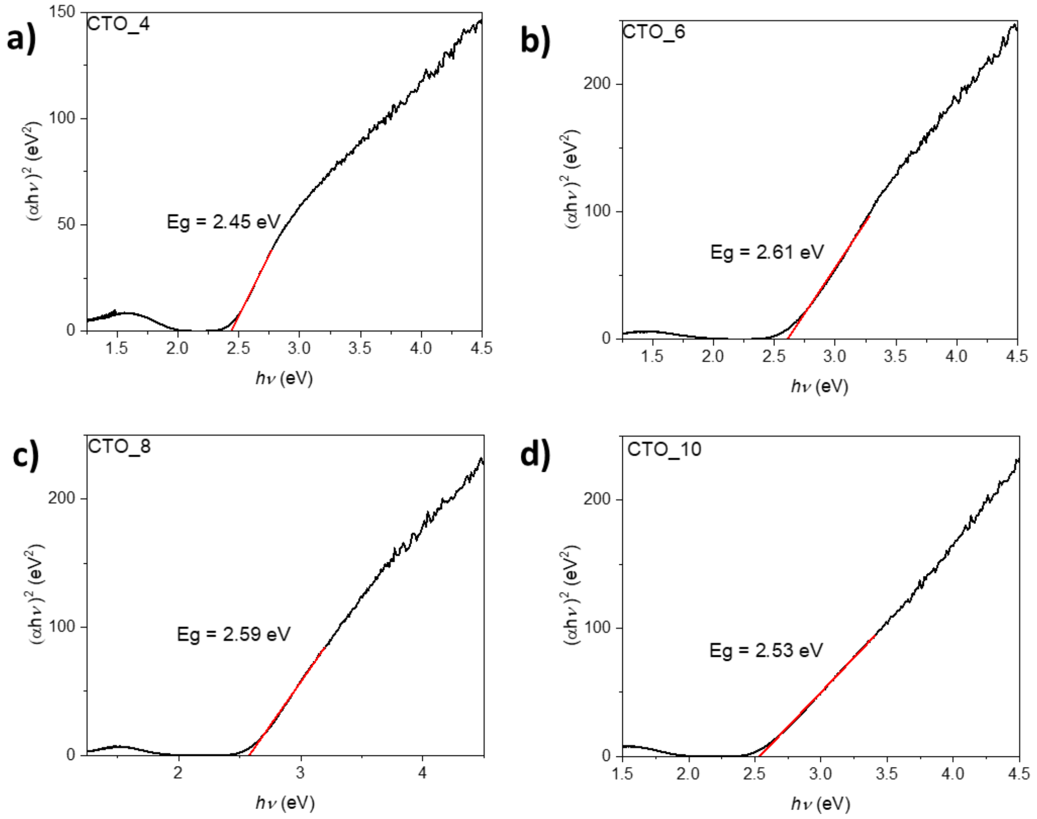

Figure S5. *Bandgap energy (Eg) calculation from the Tauc plot of the materials prepared in this work. a) CTO_4, b) CTO_6, c) CTO_8, and d) CTO_10.*

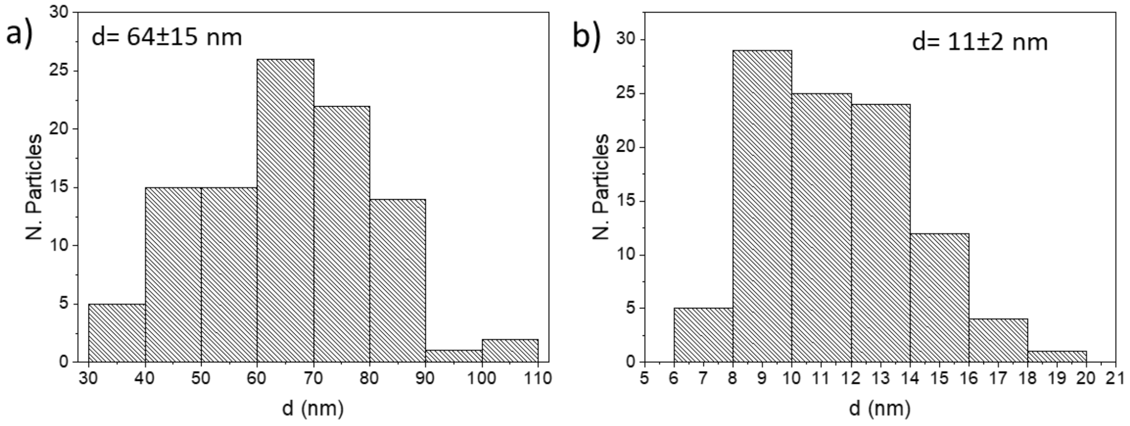

Figure S6. Size distribution *of 100 nanoparticles of a) CTO_8_Calc and b) CTO_120_6 samples.*

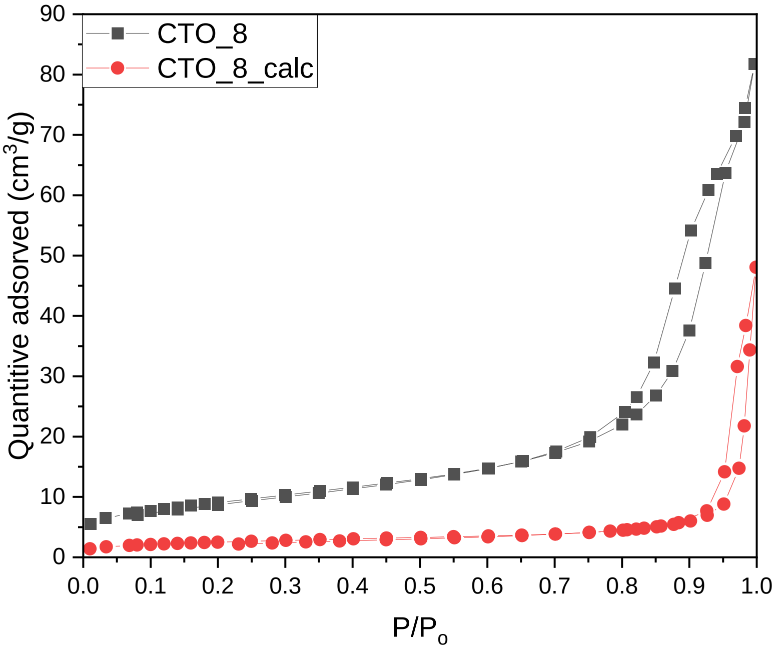

Figure S7. *N_2 isotherms at 77 K for CTO prepared without (CTO_8) and with calcination step (CTO_8_Calc).*

Table S2. *Textural properties of the CTO prepared without (CTO_8) and with calcination step (CTO_8_Calc).*

**Sample**	**S_{BET} (m^2/g)**	**$V_{total,0.95}$ (cm^3/g)**	**V_{N2DR} (cm^3/g)**
CTO_8	32.11	0.112	0.029
CTO_8_Calc	8.75	0.053	0.0055

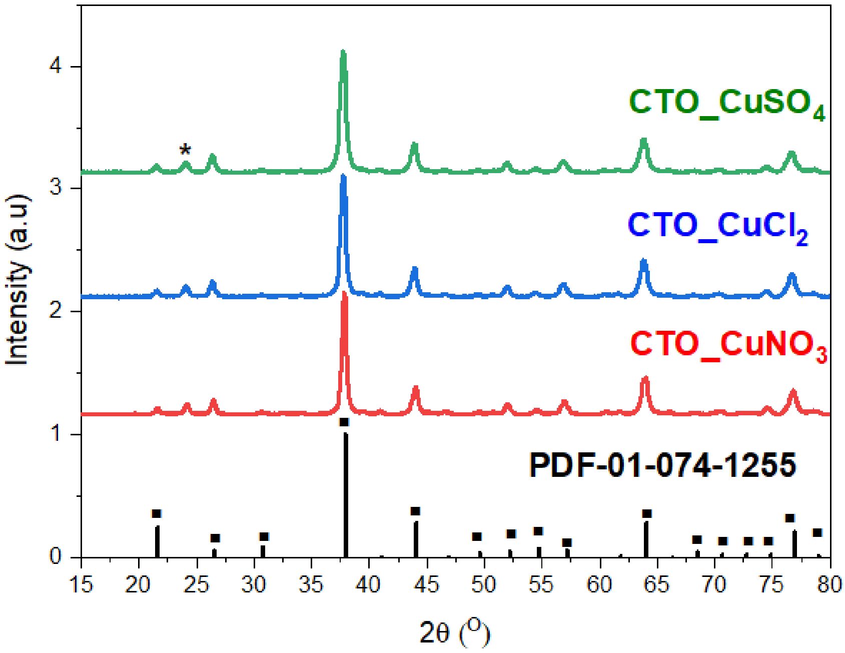

Figure S8. *XRD patterns of CTO prepared with different Cu precursors: a) $CuNO_3$, b) $CuCl_2$, and c) $CuSO_4$.*

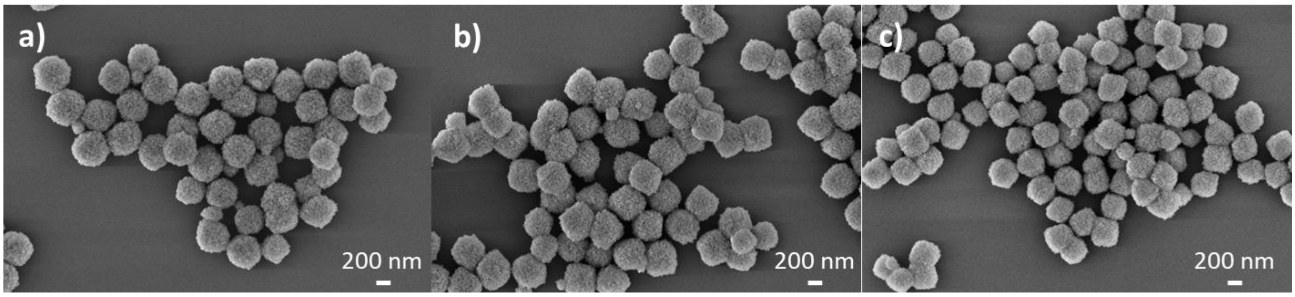

Figure S9. *SEM micrographs of CTO prepared with different Cu precursors: a) $CuNO_3$, b) $CuCl_2$, and c) $CuSO_4$.*
